# Single‐cell RNA sequencing integrated with bulk RNA sequencing analysis reveals the protective effects of lactate‐mediated lactylation of microglia‐related proteins on spinal cord injury

**DOI:** 10.1111/cns.70028

**Published:** 2024-09-01

**Authors:** Bin Zhang, Fudong Li, Yangyang Shi, Chenglong Ji, Qingjie Kong, Kaiqiang Sun, Xiaofei Sun

**Affiliations:** ^1^ Department of Orthopedic Surgery, Shanghai Changzheng Hospital Navy Medical University Shanghai China; ^2^ Department of Orthopedics, Shanghai General Hospital Shanghai Jiao Tong University School of Medicine Shanghai China; ^3^ Department of Orthopedics Naval Medical Center of PLA Shanghai China

**Keywords:** glycolysis, lactate, Lactylation, microglial polarization, neuroinflammation, spinal cord injury

## Abstract

**Background and Objectives:**

Spinal cord injury (SCI) results in significant neurological deficits, and microglia play the critical role in regulating the immune microenvironment and neurological recovery. Protein lactylation has been found to modulate the function of immune cells. Therefore, this study aimed to elucidate the effects of glycolysis‐derived lactate on microglial function and its potential neuroprotective mechanisms via lactylation after SCI.

**Methods:**

Single‐cell RNA sequencing (scRNA‐seq) data were obtained from figshare to analyze cellular and molecular alterations within the spinal cord post‐SCI, further focusing on the expression of microglia‐related genes for cell sub‐clustering, trajectory analysis, and glycolysis function analysis. We also evaluated the expression of lactylation‐related genes in microglia between day 7 after SCI and sham group. Additionally, we established the mice SCI model and performed the bulk RNA sequencing in a time‐dependent manner. The expression of glycolysis‐ and lactylation‐related genes was evaluated, as well as the immune infiltration analysis based on the lactylation‐related genes. Then, we investigated the bio‐effects of lactate on the inflammation and polarization phenotype of microglia. Finally, adult male C57BL/6 mice were subjected to exercise first to increase lactate level, before SCI surgery, aiming to evaluate the protective effects of lactate‐mediated lactylation of microglia‐related proteins on SCI.

**Results:**

scRNA‐seq identified a subcluster of microglia, recombinant chemokine C‐X3‐C‐motif receptor 1^+^ (CX3CR1^+^) microglia, which is featured by M1‐like phenotype and increased after SCI. KEGG analysis revealed the dysfunctional glycolysis in microglia after SCI surgery, and AUCell analysis suggested that the decreased glycolysis an increased oxidative phosphorylation in CX3CR1^+^ microglia. Differential gene analysis suggested that several lactylation‐related genes (Fabp5, Lgals1, Vim, and Nefl) were downregulated in CX3CR1^+^ microglia at day 7 after SCI, further validated by the results from bulk RNA sequencing. Immunofluorescence staining indicated the expression of lactate dehydrogenase A (LDHA) in CX3CR1^+^ microglia also decreased at day 7 after SCI. Cellular experiments demonstrated that the administration of lactate could increase the lactylation level and inhibit the pro‐inflammatory phenotype in microglia. Functionally, exercise‐mediated lactate production resulted in improved locomotor recovery and decreased inflammatory markers in SCI mice compared to SCI alone.

**Conclusions:**

In the subacute phase of SCI, metabolic remodeling in microglia may be key therapeutic targets to promote nerve regeneration, and lactate contributed to neuroprotection after SCI by influencing microglial lactylation and inflammatory phenotype, which offered a novel approach for therapeutic intervention.

## Graphical abstract

In the acute phase of spinal cord injury, glycolysis is weakened, and at the same time, lactic acid modification is weakened. After lactate supplementation through exercise, lactylation is increased and the motor function of mice is improved.

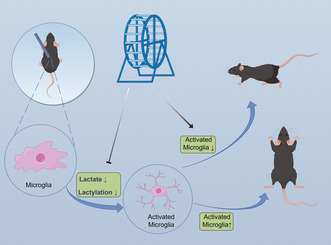

## 1 INTRODUCTION

Spinal cord injury (SCI) represents a devastating condition with profound implications for affected individuals, their families, and society. Epidemiologically, SCI exhibits a bimodal distribution, affecting predominantly young adults due to traumatic incidents such as vehicular accidents, falls, and sports‐related injuries, while also impacting older adults due to degenerative conditions and medical procedures.^1^ The consequences of SCI are multifaceted, extending far beyond the initial trauma. Patients often experience profound impairments in motor, sensory, and autonomic functions, leading to substantial decrements in quality of life.^2^ Mobility limitations, pain, bladder and bowel dysfunction, sexual dysfunction, and increased susceptibility to secondary complications such as pressure ulcers and respiratory infections further compound the challenges faced by individuals living with SCI.^3^

Pathologically, SCI initiates a cascade of events characterized by primary mechanical injury followed by secondary injury mechanisms, including neuroinflammation.^4^ SCI can be divided into three phases: acute (<48 h), subacute (48 h–2 weeks), and intermediate and chronic phases (2 weeks–6 months).^5^ During the acute phase, primary mechanical injury occurs, leading to immediate tissue damage. In the subacute phase, neuronal loss and axon and myelin necrosis activate macrophages to release pro‐inflammatory cytokines, resulting in an extensive inflammatory response within the SCI microenvironment.^6^ This phase is marked by the destruction of the blood–spinal cord barrier, which disrupts the immune microenvironment and hinders regeneration of the injured spinal cord.^7^ Central to this inflammatory response are microglia, the resident immune cells of the central nervous system.^8^ Upon activation, microglia undergo morphological and functional changes, releasing pro‐inflammatory cytokines, chemokines, and reactive oxygen species,^9^ which exacerbate tissue damage and contribute to neuronal degeneration. The interplay between microglia and neuroinflammation in the context of SCI underscores the intricate balance between detrimental and reparative processes. Microglia can be divided into M1 and M2 microglia. M1 microglia exert pro‐inflammatory activity, while M2 microglia have an anti‐inflammatory function.^10^ As SCI progresses to the intermediate and chronic phases, the role of microglia becomes increasingly complex. While microglia play a crucial role in clearing cellular debris and promoting tissue repair, their dysregulated activation can perpetuate neuroinflammatory cascades, exacerbating neuronal injury and functional deficits.^11^ Homeostatic microglia promote neuronal development, axonal regeneration, and pruning of neuronal/synaptic side branches. However, in the disease microenvironment, microglia transform into disease‐associated microglia (DAM).^12^ In SCI, DAM can be classified as inflammatory, dividing, and migrating microglia based on single‐cell RNA sequencing (scRNA‐seq) and flow cytometry.^13^ Understanding the heterogeneity of the immune microenvironment and targeting macrophages/microglia may be crucial for establishing optimal regeneration strategies after SCI. For example, targeting specific signaling pathways involved in the activation of pro‐inflammatory microglia, such as the NF‐κB pathway, could help mitigate the detrimental effects of inflammation while preserving or enhancing the beneficial functions of these cells.^14^ Additionally, modulating the balance between M1 and M2 phenotypes through therapeutic interventions, such as pharmacological agents or gene therapy, holds the potential for improving outcomes after SCI.^15^

Glycolysis, the metabolic pathway by which glucose is converted into pyruvate, plays a crucial role in SCI pathophysiology.^16^ Following SCI, glycolytic activity initially decreases during the acute phase due to the disruption of metabolic homeostasis and energy production.^17^ This reduction in glycolysis hampers the energy supply needed for cellular repair and immune response, exacerbating tissue damage and neuronal degeneration. This decreasing level of glycolysis leads to lowered production of lactate, a byproduct of glucose metabolism.^18^ Dysregulated glycolysis and lactate metabolism contribute to inflammation and exacerbate cell damage,^19^ highlighting the potential therapeutic importance of targeting these metabolic pathways in SCI. Lactate, a byproduct of glycolysis, plays a significant role in modulating the inflammatory response, particularly in the context of SCI. Lactate has been reported to suppress the pro‐inflammatory response of macrophage stimulated by lipopolysaccharide (LPS) via G protein‐coupled receptor 81 (GPR81)‐mediated signaling, reducing the production of pro‐inflammatory cytokines. Additionally, lactate could induce M2 macrophage polarization in breast cancer, which helps create an anti‐inflammatory environment.^20^ This polarization is further supported by the functional polarization of tumor‐associated macrophages by tumor‐derived lactic acid, emphasizing lactate's role in promoting anti‐inflammatory macrophage phenotypes. Furthermore, lactate‐driven macrophage polarization in the inflammatory microenvironment has been shown to alleviate intestinal inflammation, demonstrating its broad anti‐inflammatory potential. In the context of SCI, lactate's anti‐inflammatory properties may help mitigate neuroinflammation, protect neuronal tissue, and promote healing, making it a potential therapeutic target for enhancing neuroprotection and recovery following injury.^21^ Specifically, lactate serves as a substrate for lactylation, a recently discovered post‐translational modification with profound implications for immune regulation and tissue repair processes.^22^ Lactylation has been reported to function in multiple physiological and pathological processes. Wang et al. reported that lactate increased the lactylation level of pyruvate kinase isozymes M2 (PKM2), promoting its pyruvate kinase activity.^23^ In addition, several studies have highlighted the role of lactylation in inflammation, emphasizing its potential as a therapeutic target.^24^ Modulating lactylation might be a promising avenue for mitigating neuroinflammation and enhancing neuroprotection in SCI. Moreover, the interplay between metabolic pathways and immune responses is critical in the context of SCI. Metabolic reprogramming of immune cells, including microglia, influences their activation and function. For instance, the shift from oxidative phosphorylation to glycolysis, known as the Warburg effect, is associated with the pro‐inflammatory activation of immune cells.^25^ Conversely, promoting oxidative metabolism can support anti‐inflammatory phenotypes.^26^ Targeting these metabolic shifts in immune cells holds the potential for modulating inflammation and improving outcomes in SCI. However, currently, there are limited studies exploring lactylation‐related genes and their role in SCI.

Therefore, based on an integrated analysis of single‐cell RNA sequencing and bulk RNA sequencing analysis, this present study aimed to elucidate the role of glycolysis‐derived lactate in modulating microglial function and attenuating SCI and to delineate the molecular mechanisms underlying lactylation‐mediated neuroprotection. By elucidating these mechanisms, we aspired to not only advance our fundamental understanding of SCI pathology but also to pave the way for the development of targeted therapeutic interventions for SCI.

## 2 METHODS

### 2.1 Acquisition and analysis single‐cell RNA sequencing data

Single‐cell RNA sequencing data were retrieved from figshare with the identifier (https://doi.org/10.6084/m9.figshare.17702045).^27^ The Seurat package was used to process scRNA‐seq data. Briefly, we removed low‐quality data, as barcodes with outlier gene counts may be indicative of dying cells, cells with broken membranes, or doublets. We also removed cells in which >20% of unique molecular identifiers mapped to mitochondrial genes because a very high fraction of mitochondria is indicative of leaky cytoplasmic membranes allowing mRNA to escape. After the removal of low‐quality cells, the remaining high‐quality cells were normalized and scaled in order to acquire linear conversion by using the “NormalizeData” function and the “ScaleData” function. The top 2000 variable genes were selected for principal component analysis (PCA), and the 30 most important principal components were selected for cluster analysis. Using the “IntegratedLayers” function, batch effects were eliminated by combining scRNA‐seq data from several samples. The clusters were then visualized by means of Uniform Manifold Approximation and Projection (UMAP). Cells were grouped based on the expression of three classical markers: Endothelial cell (Flt1, Ly6c1, Cldn5); Microglia (C1qa, C1qb, Ctss); Leukocyte (Ccl5, Ma4a4b, AW112010); Erythrocyte (Hbb‐bs, Hba‐a1, Hba‐a2); Astrocyte (Aldoc, Slc4a4, Atp1b2); Stromal cell (Apod, Dcn, Ptgds); Pericyte (Vtn, Rgs5, Cald1); Neutrophil (S100a8, S100a9, Cxcl12); Neuro (Meg3, Snhg11, Kcnq1ot1); Ependyma (Nant, 1500015O10Rik, Rarres2); ODC (Vcan, Cspg5, Olig1); and OPC (Plp1, Mbp, Mpz). A total of 332 lactylation‐related genes were included in this study according to a previous study.^28^

### 2.2 Trajectory analysis

Monocle 2 or cell differentiation trajectory analysis was used. This analysis commenced with data normalization to mitigate technical biases, followed by differential expression testing to identify genes marking cellular transitions. Utilizing Monocle 2's unsupervised learning, we constructed cellular trajectories, revealing the dynamism of differentiation within microglia subsets. The pseudo‐time‐dependent gene expression across all microglia was further identified using an unsupervised approach.^29^

### 2.3 GO and KEGG enrichment analysis

The R package clusterProfiler conducted Gene Ontology (GO) and Kyoto Encyclopedia of Genes and Genomes (KEGG) annotation analysis on common differential genes,^30^ with entry selection criteria of *p* value <0.05 and FDR value (*q* value) < 0.05 deemed statistically significant. The top 30 pathways were selected based on *p*‐value ranking.

### 2.4 Score according to metabolism‐related hallmark genes

To calculate module scores and the fraction of enrichment for metabolism‐related gene expression and M1/2 score in single cells, AUCell analysis was performed.^31^ The glycolysis‐ and oxidative phosphorylation‐related hallmark genes or M1/2 genes were downloaded from the gene set enrichment analysis (GSEA) database (https://www.gsea‐msigdb.org/).^32^ We calculated the glycolysis and oxidative phosphorylation score and AUC value in each cell type with the metabolism‐related hallmark genes.

### 2.5 The procedure of establishing SCI model

All experimental protocols were approved by the Ethics Committee of Shanghai Changzheng Hospital. Adult male C57BL/6 mice, aged 6–8 weeks and weighing 20–25 grams, were procured from the Experimental Animal Center of Zhejiang Academy of Medicinal Sciences, Hangzhou Medical College, Hangzhou, China. These animals were allocated into distinct groups: sham, SCI, and exercise prior to SCI. SCI was induced following a previously established method.^14^ Anesthesia was administered using isoflurane (2%–3% for induction and 1%–2% for maintenance) to ensure the mice remained pain‐free and immobile during the procedure. The back of each mouse was shaved and disinfected, and sterile drapes were used. A midline incision was made at the thoracic region to expose the T10 vertebra, followed by a laminectomy to reveal the spinal cord. SCI was inflicted using a weight‐drop device (model 68,097, RWD, CA, USA), where a 5‐gram rod was dropped from a height of 5 cm onto the exposed spinal cord, resulting in a reproducible contusion injury. After injury, the muscle and skin layers were sutured with 4–0 silk sutures, and the mice were placed on a heating pad to maintain body temperature during recovery. In the sham group, a laminectomy was performed without the weight‐drop impact, serving as the control. Post‐operative care included administering analgesics, providing hydration, and monitoring for signs of distress or complications, with bladder expression performed twice daily until spontaneous voiding was observed. This detailed methodology ensures the reproducibility and consistency of the SCI model for valid experimental outcomes.

### 2.6 Bulk sequencing data processing

mRNA‐seq analysis of mice spinal cord tissue at different time points was performed from our laboratory with the help of Genekinder Medicaltech (Genekinder Medicaltech (Shanghai) Co., Ltd，China). Then, the R package Deseq2 was used to analyze differentially expressed genes (DEGs). Additional analysis was conducted on mRNAs that were differentially expressed and showed a log2(FC) value >1.5 and *p* value <0.05. An illustration of DEGs between two samples was shown in volcano and heatmap plots by using ggplot2 and heatmap packages.

### 2.7 Basso mouse scale

The Basso Mouse Scale (BMS) is an evaluative tool designed to measure locomotor recovery in mice following SCI. It employs a 0–9 scoring system, where a score of 0 indicates complete paralysis with no observable leg movement, and a score of 9 signifies normal locomotion. The BMS assesses various aspects of motor function, including hindlimb movement, joint movements, stepping ability, trunk stability, and coordination during open‐field locomotion.^33^

### 2.8 Swimming score

The swimming score is a specialized metric used to assess the recovery of motor function in mice following SCI through their performance in a swimming test. This scoring system evaluates several key aspects of motor abilities, including the use of hindlimbs, coordination and symmetry of movements, tail position, and the overall effort and endurance of the animal.^34^ The scale ranges from a complete inability to swim, marked by a lack of hindlimb movement and coordination, to proficient swimming characterized by effective, coordinated use of all limbs and the tail for propulsion and balance.

### 2.9 Terminal deoxynucleotidyl transferase dUTP nick end labeling staining

Seven days following injury, tissue sections were cryopreserved and processed. Apoptosis was detected using the terminal deoxynucleotidyl transferase dUTP nick end labeling (TUNEL) assay, according to the manufacturer's protocol. This method entails the enzymatic incorporation of marker‐labeled dUTP at the 3'‐OH termini of DNA fragments by terminal deoxynucleotidyl transferase. The labeled DNA fragments were then visualized under a fluorescence microscope, enabling accurate identification and quantification of apoptotic cells in the spinal cord tissue adjacent to the injury epicenter. Counts of TUNEL‐positive cells were recorded.

### 2.10 Immunofluorescent staining

BV2 cells were fixed to preserve cellular structures and immobilize the proteins, which were then permeabilized with Triton X‐100. Cells were blocked with bovine serum albumin to prevent non‐specific binding of the antibodies to the cells. The immunofluorescent staining procedure of spinal cord tissues began with the careful preparation of spinal cord sections, including fixation with paraformaldehyde to preserve tissue architecture and protein integrity. After fixation, the tissue was embedded in paraffin and sectioned into thin slices for staining. These sections were then subjected to antigen retrieval processes to unmask epitopes. Following antigen retrieval, the sections are blocked to prevent non‐specific binding of antibodies to the tissue. The cells or sections were then incubated with primary antibodies at 4°C overnight, which included CX3CR1 (Cat#DF7096, Affinity), LDHA (Cat#DF6280, Affinity), cluster of differentiation 206 (CD206, Cat# 24595, Cell signaling technology), inducible nitric oxide synthase (iNOS, Cat#ab178945, Abcam), ionized calcium binding adaptor molecule‐1 (IBA‐1, Cat# ab178846, Abcam), and Pan‐Kla (Cat# PTM‐1401RM, Jingjie PTM BioLab Co., Ltd). After washing away any unbound primary antibodies, the cells or sections were incubated with fluorescently labeled secondary antibodies. Finally, the cells or sections were washed to remove unbound secondary antibodies and counterstained with DAPI to visualize cell nuclei.

### 2.11 Nissl's staining

The fixed spinal cord tissue was embedded in paraffin wax, enabling thin sections to be cut for staining. These sections were then mounted on slides. Before staining, the paraffin was removed with xylene, and the sections were rehydrated through a graded alcohol series. The slides were then stained with the Nissl stain (Cat#G1036, Servicebio), where the dye binds to the nucleic acids present in the neuron's cell body, particularly highlighting the rough endoplasmic reticulum and nucleolus, which are abundant in neurons. After staining, the slides are dehydrated, cleared, and mounted for microscopic examination.

### 2.12 Lactate detection

Lactate production was measured using a Lactate Assay Kit (Cat#BC2235, Beijing Solarbio Technology Co., Ltd), as described by the protocol. One mL of extract I was added to the cells of the pretreated 96‐well plates, and the cells were broken by ultrasound in an ice bath (ultrasonic for 3 s, interval for 7 s, total time for 3 min). After centrifugation at 12000 *g* for 10 min at 4°C, 0.8 mL of the supernatant was taken, and 0.15 mL of extract II was added, and the supernatant was taken after centrifugation at 12000 *g* for 10 min at 4°C for measurement. The microplate reader was preheated for more than 30 min, and the wavelength was adjusted to 570 nm. The standard solution of 100 μmol/mL was diluted with distilled water to 2.5, 1.25, 0.625, 0.3125, 0.15625, and 0.078 μmol/mL standard solution to be measured.

### 2.13 Reverse transcription polymerase chain reaction

The reverse transcription polymerase chain reaction (RT‐PCR) method was employed to assess gene expression in specified tissues or cells. Initially, spinal cord tissues were collected from mice, with precautions taken to prevent RNA degradation via rapid freezing or the application of RNA stabilizing agents. RNA extraction from these tissues involved homogenization followed by chemical separation techniques to ensure RNA purity and integrity for subsequent use. This RNA then served as a template for synthesizing complementary DNA (cDNA) through reverse transcription, facilitated by the reverse transcriptase enzyme using HiScript II Q RT SuperMix (R122‐01, Vazyme, China). The resultant cDNA was amplified by PCR using specific primers targeting genes of interest. The PCR amplification process involved an initial denaturation step at 95°C for 3 min, followed by 40 cycles of denaturation at 95°C for 10 s, annealing at 60°C for 30 s, and extension at 72°C for 30 s. This amplification was monitored in real‐time through quantitative RT‐PCR (qRT‐PCR) using ChamQ qPCR SYBR Green Master Mix (Q111‐02, Vazyme, China). Relative gene expression levels were quantified by normalizing against GAPDH using the 2^−ΔΔCT^ method, with primer sequences detailed in Table 1.

**TABLE 1:** Primers of the genes used in the study.

Gene	Primer sequences
IL‐1β	5’‐GAACAACAAAAATGCCTCGTGC‐3’ (forward)
	5’‐ TGTCGTTGCTTGTCTCTCCTTGT‐3’ (reverse)
iNOS	5’‐GGAGTGACGGCAAACATGACT‐3’ (forward)
	5’‐TCGATGCACAACTGGGTGAAC‐3’ (reverse)
TNF‐α	5’‐GGGTGATCGGTCCCCAAAGG‐3’ (forward)
	5’‐TTGAGAAGATGATCTGAGTGTGAGG‐3’ (reverse)
Arg‐1	5’‐TTGGGTGGATGCTCACACTG‐3’ (forward)
	5’‐GTACACGATGTCTTTGGCAGA‐3’ (reverse)
CD206	5’‐CAAGCGATGTGCCTACC‐3’ (forward)
	5’‐AATGCTGTGGATACTTGCC‐3’ (reverse)
IL‐10	5’‐TGCTATGCTGCCTGCTCTTA‐3’ (forward)
	5’‐TCATTTCCGATAAGGCTTGG‐3’ (reverse)
β‐Actin	5’‐CAGCCTTCCTTCTTGGGTAT‐3’ (forward)
	5’‐TGGCATAGAGGTCTTTACGG‐3’ (reverse)

### 2.14 Western blotting

BV2 cells were harvested and lysed using a protein extraction buffer at 4°C. Protein concentrations were determined by the bicinchoninic acid (BCA) assay. Subsequently, the proteins were separated by electrophoresis and transferred to polyvinylidene fluoride (PVDF) membranes. These membranes were blocked using 5% nonfat skimmed milk for 2 h at ambient temperature before overnight incubation at 4°C with primary antibodies: interleukin‐1 beta (IL‐1β, Cat#ab283818, Abcam); iNOS (Cat#ab178945, Abcam); tumor necrosis factor‐alpha (TNF‐α) (Cat#ab283818, Abcam); arginase‐1 (Arg‐1) (Cat# 93668, Cell Signaling Technology); CD206 (Cat# 24595, Cell Signaling Technology); interleukin‐10 (IL‐10) (Cat# ab189392, Abcam); and β‐actin (Cat# ab6276, Abcam). Secondary antibodies were applied at a 1:5000 dilution and incubated for 2 h at room temperature. Detection of immunoreactive bands was achieved using enhanced chemiluminescence (ECL) reagents (PE0010, Solarbio, Beijing, China), and the band densities, indicative of protein quantities, were quantified utilizing ImageJ software.

### 2.15 Statistical analyses

All experiments were conducted in triplicate or more. Data analysts were blinded to the identities of the control and treatment groups to mitigate bias. Statistical analysis was performed using SPSS software version 26.0 (Chicago, USA). Results were expressed as mean ± standard deviation. All data were subjected to formal tests for normality, specifically the Shapiro–Wilk test. Quantitative data across multiple groups were compared via analysis of variance (ANOVA) if they followed a normal distribution, whereas comparisons between two groups employed the *t*‐test. For data that did not exhibit a normal/Gaussian distribution, non‐parametric equivalents such as the Kruskal–Wallis test (to compare the distributions of three or more independent groups) and the Mann–Whitney *U* test (to compare the distributions of two independent samples) were used. Statistical significance was established at P values less than 0.05.

## 3 RESULTS

### 3.1 Molecular identification of immune, vascular, and glial cells in the SCI site

**FIGURE 1:**
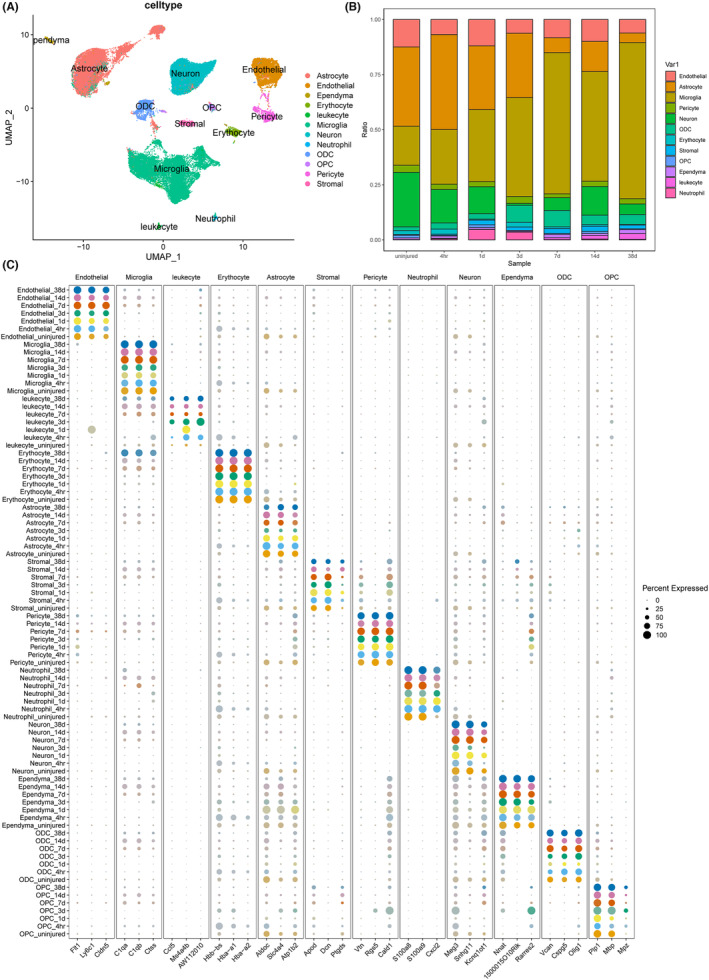
Molecular identification of various cells in the SCI site. (A) UMAP plot of all cells collected from the uninjured spinal cord and injured spinal cord. (B) Relative frequency of cell clusters at each time point. (C) Dot plot of highest DEGs for each major cell type at each time point.

After integrating data from the uninjured group (33,285 cells), the 4‐hour group after SCI (3401 cells), the 3‐dpi group (936 cells), the 7‐dpi group (6237 cells), the 14‐dpi group (9449 cells), and the 38‐dpi group (3800 cells), a total of 59,558 cells were identified. The single‐cell atlas of the spinal cord following injury is presented in Figure 1. Cluster analysis of these cells resulted in 12 distinct clusters including astrocyte, endothelial cell, ependyma cell, erythrocyte, leukocyte, microglia, neuron, neutrophil, stromal cell, pericyte, oligodendrocyte (ODC), and oligodendrocyte progenitor cells (OPC) with specific temporal progression when visualized on a uniform manifold approximation and projection (UMAP) plot (Figure 1A). The results showed that various cell types were identified, including neurons, astrocytes, oligodendrocyte precursor cells (OPCs), microglia, endothelial cells, and peripheral immune cells such as neutrophils (Figure 1A). A stacked bar chart represented the proportion of the various cells across time points, indicating the dynamic changes in cellular composition during the course of recovery. The results showed that the percentage of microglia increased remarkably after SCI (Figure 1B). A dot plot presented the expression levels of key genes across identified cell types, providing insights into cell‐specific gene expression changes post‐injury, such as upregulation of C1qa, C1qb, and Ctss in microglia (Figure 1C). While certain marker genes were expressed both before and after injury, others, such as C1qa, C1qb, and Ctss in microglia, changed expression in an injury‐dependent manner. While C1qa, C1qb, and Ctss of microglia were highly expressed in uninjured mice, there was a graded decrease at the acute and subacute phases. Aldoc, Slc4a4, and Atp1b2 were remarkably lowered on the 3rd day and the 7th day when compared with those before surgery, on 14 days, and 38 days (Figure 1C).

### 3.2 Subcluster analysis identified CX3CR1 ^+^ microglia as the critical subgroup related to the inflammatory microenvironment after SCI

**FIGURE 2:**
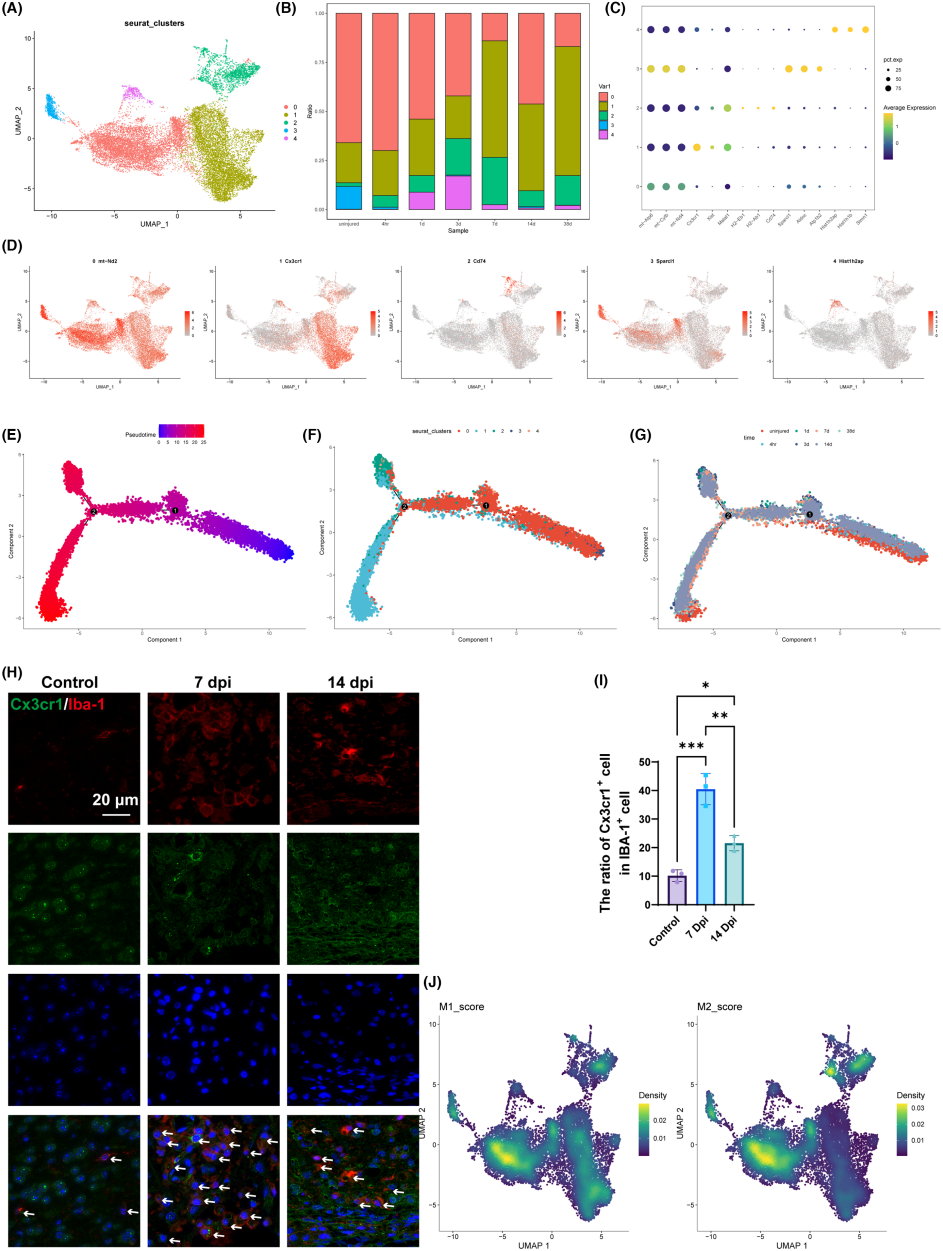
Cx3cr1+ microglia might be associated with M1 pro‐inflammatory activity. (A) UMAP plot of microglia clusters. (B) The ratio of different microglia clusters at each time point. (C) The expression characteristics of different genes in microglia clusters. (D) The visualization results of UMAP analysis for different genes. (E) Pseudotime trajectories of all single cells according to bifurcation 1 and bifurcation 2. (F) The results of pseudotime trajectory analysis for different microglia clusters. (G) The results of pseudotime trajectory analysis at different time points after surgery. (H) Immunofluorescence results of Iba‐1 and Cx3cr1 in spinal cord samples. (I) Quantitative analysis of the immunofluorescence results. (J) The UMAP analysis of M1‐like and M2‐like microglia.

A multi‐dimensional single‐cell analysis of microglia from a mouse model of spinal cord injury (SCI) was carried out. The UMAP plot categorized microglia into distinct clusters based on gene expression profiles, suggesting heterogeneity within the microglial response to SCI (Figure 2A). The distribution of microglial subtypes across different stages post‐injury was presented in the bar chart, indicating a temporal change in the microglial landscape (Figure 2B). The bubble diagram correlated gene expression levels with microglia clusters, showing that mt‐Atp6, mt‐Cytb, and mt‐Nd4 were expressed in different microglial clusters and that Cx3cr1, Cd74, Sparcl1, and Hist1h2ap, were highly expressed in cluster 1, cluster 2, cluster 3, and cluster 4, respectively (Figure 2C). The UMAP analysis of different genes was visualized with the above results (Figure 2D). Microglia respond to injurious stimuli by switching from one functional “state” to another. Cells in different states express different genes. As cells transition between states, they undergo a process of transcriptional reorganization in which some genes are silenced, while others are activated. Pseudotime trajectory analysis module was implemented for reordering cells based on the expression profile of highly variable genes. The tree structures of pseudotime trajectory analysis consist of 4 states and 3 branches (Figure 2E). Multiple clusters (0–4) are evident, each with distinct spatial locations along the pseudotime trajectory. Cluster 1 was mainly identified after bifurcation 2 (Figure 2F). Pseudotime trajectory analysis of microglia at different time points showed that after bifurcation 2, microglial cells at 14 days post‐injury were mainly identified (Figure 2G). Immunofluorescence images validating the UMAP and trajectory findings at the protein level showed increased Iba‐1 staining in injured versus control tissue and revealed that the Cx3cr1 intensity was raised at 7 dpi and14 dpi (Figure 2H,I). In addition, the differentially expressed genes analysis between cluster 1 and cluster 2 showed that Cx3cr1 was upregulated in cluster 1 and that neuroinflammatory response was enhanced (Figure S1–S2A,B). The distribution analysis of microglia showed that M1 microglia were specifically distributed in cluster 1 and M2 microglia in cluster 2 (Figure 2J). The above results demonstrated that Cx3cr1+ microglia in cluster 1 might present M1 pro‐inflammatory activity.

### 3.3 After spinal cord injury, the expression of lactation‐related genes was significantly decreased in CX3CR1 ^+^ microglia

**FIGURE 3:**
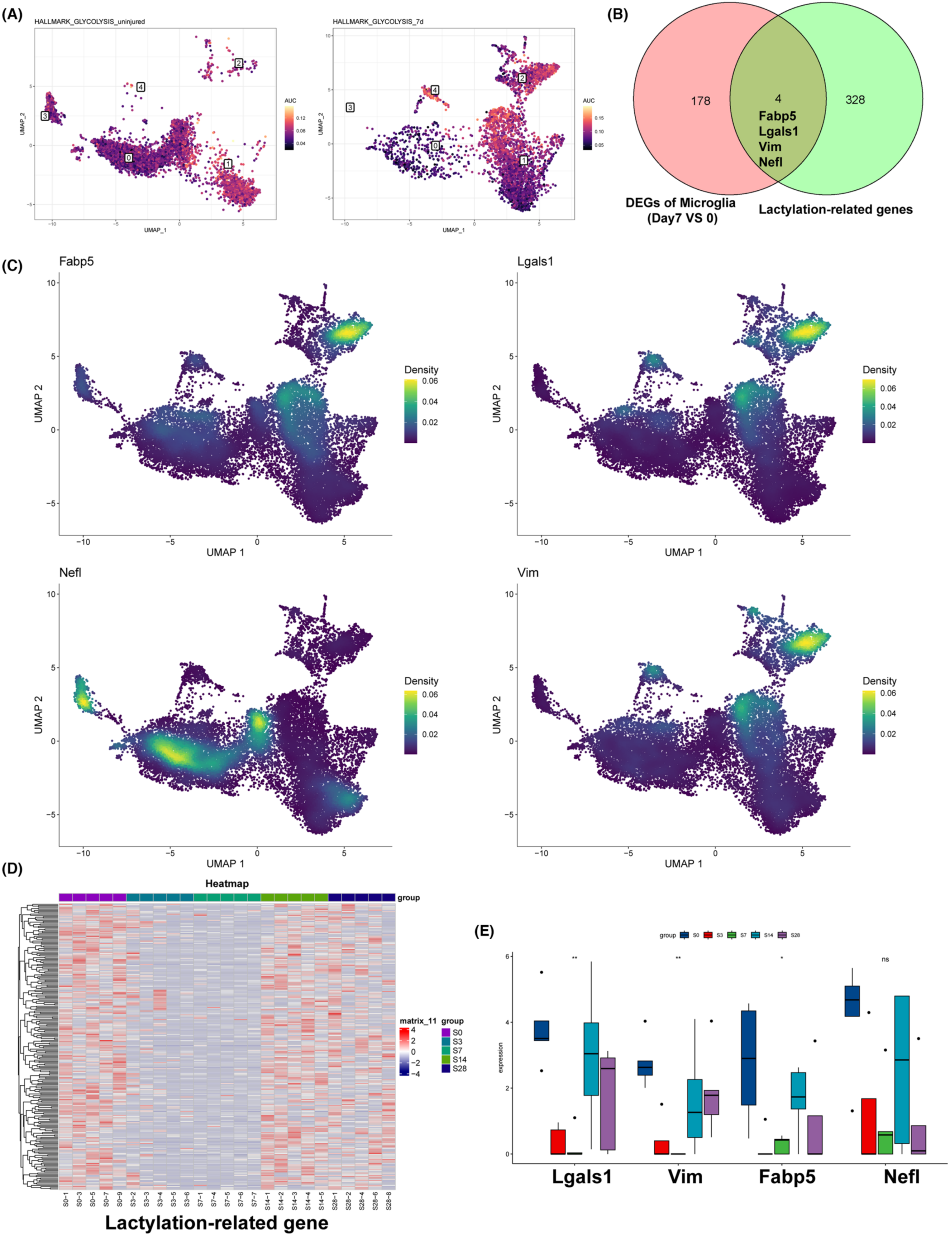
The expression of lactation‐related genes was decreased in CX3CR1+ microglia after spinal cord injury. (A) The UMAP analysis results of glycolysis score for uninjured mice and SCI mice at day 7 after injury. (B) Venn diagram of microglia DEGs and lactylation‐related genes. (C) The UMAP analysis results of genes in microglia clusters. (D) The heatmap of lactylation‐related genes. (E) Expression levels of key genes include Lgals1, Vim, Fabp5, and Nefl.

KEGG analysis showed that glycolysis was enhanced at 3 dpi and glucose metabolism was muddled at 7, 14, and 38 dpi, demonstrating a disordered glycolytic activity post‐SCI (Figure S2 A–D). In addition, the levels of glycolysis and oxidative phosphorylation in cells of spinal cord were analyzed and the results showed that glycolysis was lowered in microglia (Figure S2E). The glycolysis and oxidative phosphorylation in different clusters of microglia were also analyzed, and the results showed that cluster 2 presented higher glycolysis activity and cluster 1 presented higher oxidative phosphorylation level (Figure S2F), indicating that Cx3cr1+ microglia might present lower glycolysis activity and higher oxidative phosphorylation level post‐injury. Compared to the microglia in the uninjured mice, the proportion of glycolysis in the SCI mice at 7 dpi was remarkably lowered in the Cx3cr1+ microglia (Figure 3A). The Venn diagram of microglia DEGs and lactylation‐related genes showed four genes including Fabp5, Lgals1, Vim, and Nefl (Figure 3B), and the four genes' distribution in the microglia clusters was analyzed and presented in Figure 3C. The results revealed that Fabp5, Lgals1, and Vim were highly located in cluster 2, while Nefl was mainly located in cluster 0 and cluster 3 (Figure 3C). Additionally, transcriptomic analysis of lactate‐related genes demonstrated a considerable decrease post‐injury on days 3 and 7, with levels returning to near baseline by days 14 and 28, indicating that lactylation was suppressed in the acute and subacute phases after SCI (Figure 3D). Furthermore, the expression patterns of Fabp5, Lgals1, Vim, and Nefl were characterized at various stages post‐SCI. In comparison with the S0 (uninjured) group, decline in expressions of Fabp5, Lgals1, Vim, and Nefl was observed on days 3 and 7, followed by a significant resurgence by days 14 and 28 (Figure 3E), confirming the lowered lactylation level in the acute and subacute phases after SCI.

### 3.4 Validation of the glycolysis level in microglia in vitro

**FIGURE 4:**
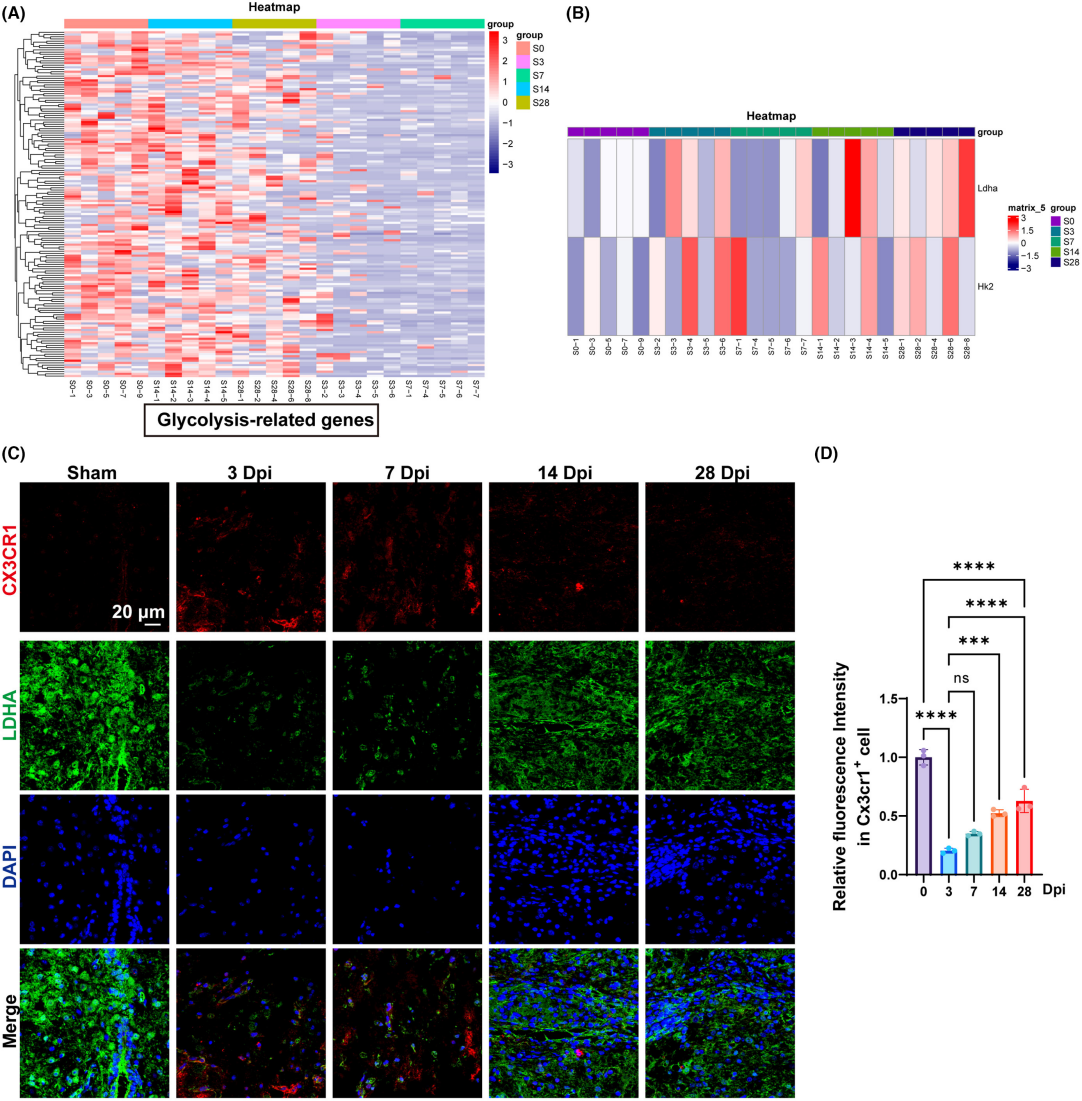
The glycolysis and lactate modification levels of microglia at different stages. (A) The heatmap results of glycolysis‐related genes. (B) The heatmap results of LDHA and HK2 at various stages. (C) The immunofluorescence results of CX3CR1 and LDHA in mice. (D) Relative immunofluorescence intensity of LDHA in Cx3cr1^+^ cells. “*,” *p* < 0.05; “**,” *p* < 0.01; “***,” *p* < 0.001; “****,” *p* < 0.0001.

To elucidate the alterations in glycolytic activity and lactate‐related gene expression in microglia following SCI, we conducted transcriptome sequencing of spinal cord tissue pre‐injury (S0), and at 3 (S3), 7 (S7), 14 (S14), and 28 (S28) days post‐SCI. Our findings reveal a significant downregulation of glycolysis‐associated genes on days 3 and 7 post‐injury compared to pre‐injury levels, with a partial restoration observed by days 14 and 28 (Figure 4A). Fluctuations in the expression of LDHA and HK2 were noted across the timeline, underscoring the disruption of glycolytic processes following SCI (Figure 4B). The immunofluorescence and semiquantitative analysis results showed that LDHA intensity in the 3 dpi and 7 dpi groups was significantly lower than that in the sham group, indicating that glycolysis in the acute and subacute group was suppressed (Figure 4C,D). However, the LDHA intensity in the 14 dpi group and the 28 dpi group was remarkably restored, when compared with that in the 3 dpi and 7 dpi groups (Figure 4C,D), showing that the glycolysis was relatively enhanced 14 days after SCI.

### 3.5 Correlation between lactylation‐related genes and immune infiltration after SCI

**FIGURE 5:**
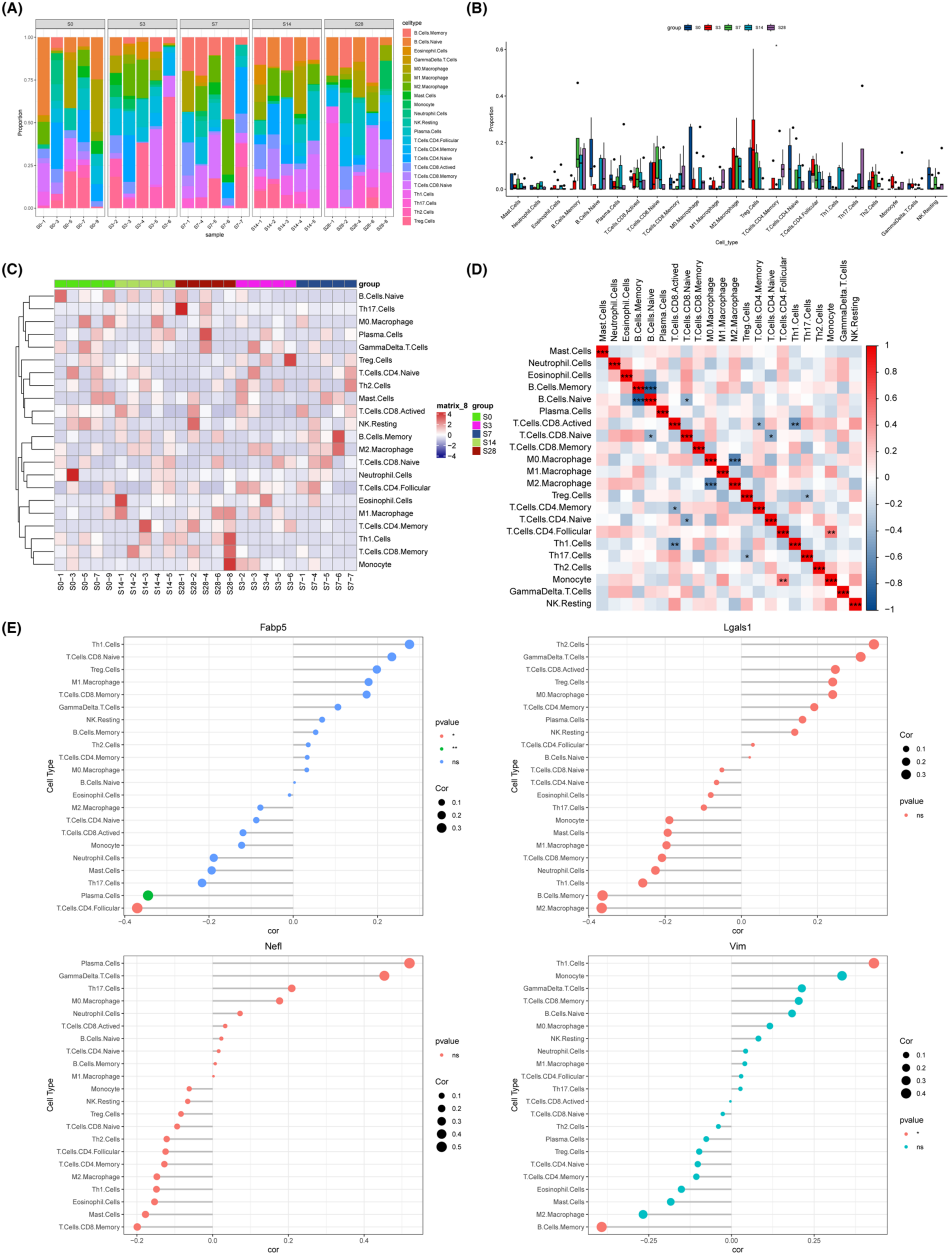
Correlation between lactylation‐related genes and immune infiltration after SCI. (A) The proportions of various immune cells at different stages. (B) The barplot of various immune cells proportions. (C) The heatmap of various immune cells at different stages. (D) The heatmap of the correlation coefficients between various immune cells. (E) The levels of Fabp5, Lgals1, Vim, and Nefl in various immune cells.

The results showed that B cells, eosinophil cells, Gamma delta T cells, macrophage, mast cells, monocyte, neutrophil cells, NK resting, plasma cells, T cells, Th cells, and Treg cells were most dominant in the injured spinal cord samples (Figure 5A). In addition, the quantitative analysis of immune cell infiltration showed that the proportions of various cells such as B cells, T cells, M1 and M2 macrophage, T cells, and NK cells were altered at different stages post‐injury (Figure 5B). The heatmap analysis revealed a heterogeneous response among immune cell populations over the course of SCI progression (Figure 5C). Certain cell types, such as M0 macrophages and neutrophils, exhibit a marked escalation in presence, indicated by pronounced red hues at specific time points, suggesting an acute inflammatory response. In contrast, subsets like naive T cells, both CD4 and CD8, appear to diminish in the early stages, as evidenced by the blue tints, potentially reflecting an initial suppression or migration away from the injury site. Memory B cells, M1 macrophages, M2 macrophages, and Th17 cells show variable infiltration levels, oscillating between increases and decreases over the studied period. The heatmap of the correlation coefficients between various types of immune cells was presented (Figure 5D). Monocyte shows strong positive correlations with follicular CD4+ T cells, reflecting their coordinated response in immune activities. Certain cells like M2 macrophage and Th1 cell subsets exhibit negative correlations with other subsets (like M0 macrophage and CDB activated T cells), indicative of the typical immune balancing act where different cell types rise in response to different cues and may suppress or be suppressed by other types. In addition, the levels of Fabp5, Lgals1, Vim, and Nefl in various immune cells in spinal cord were analyzed (Figure 5E). The results showed that Fabp5 was significantly lowered in plasma cells and CD4 T cells, and that Vim was upregulated in Th1 cells and downregulated in memory B cells. However, Lgals1 and Nefl were not significantly altered in the immune cells.

### 3.6 The administration of lactate improved the overall lactylation level and promoted M2 polarization of microglia

**FIGURE 6:**
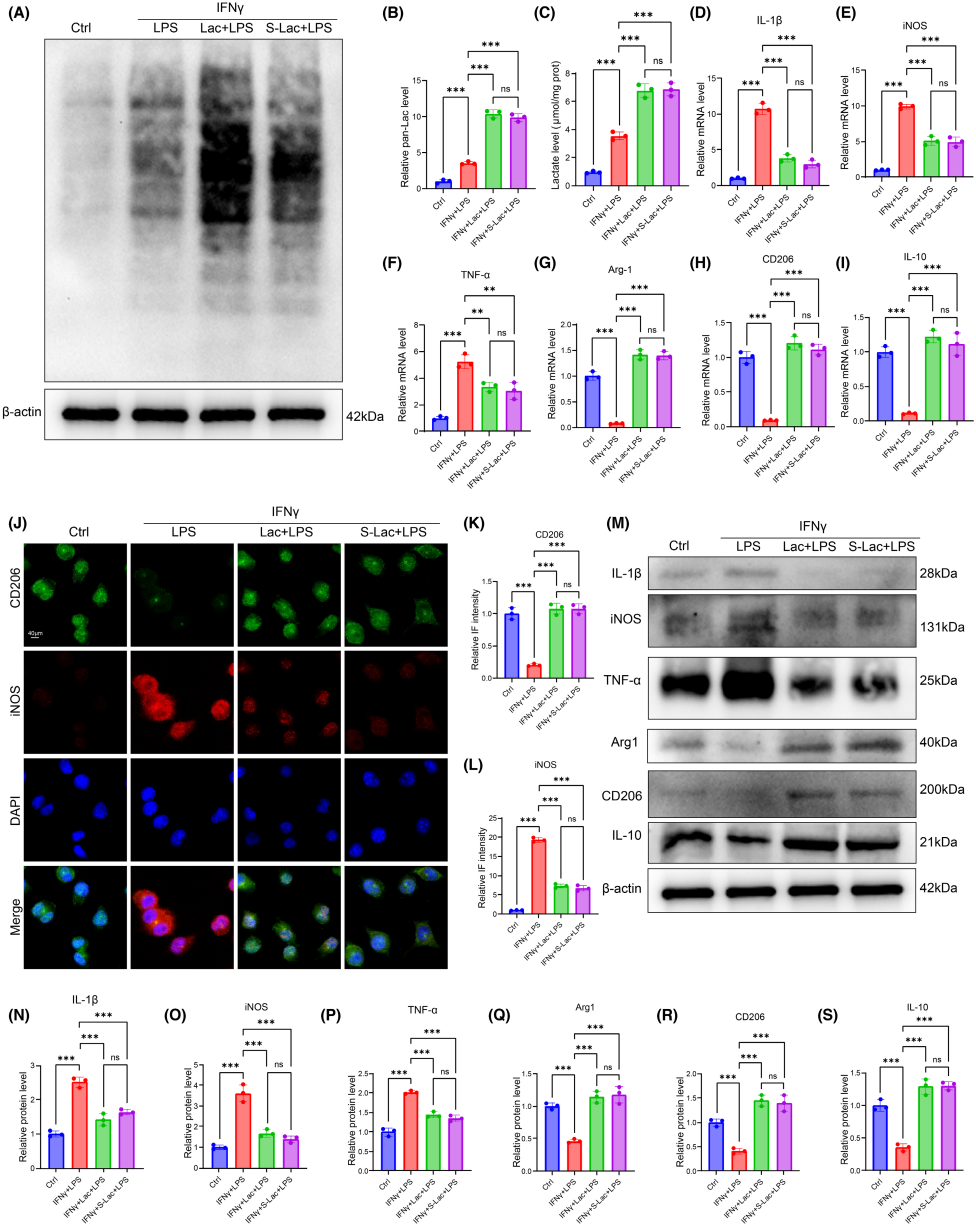
Lactate improved the overall lactylation level and promoted M2 polarization. (A) Western blot results of lactylation. (B) The semi‐quantification analysis of lactylation. (C) Lactate level in various groups. (D–I) The semi‐quantification analysis of mRNA levels of IL‐1β, iNOS, TNF‐α, Arg‐1, CD206, and IL‐10. (J) The results of immunofluorescence for CD206 and iNOS. (K,L) The semi‐quantification analysis of CD206 and iNOS. (M) Western blot results of IL‐1β, iNOS, TNF‐α, Arg‐1, CD206, and IL‐10. (N–S) The semi‐quantification analysis of IL‐1β, iNOS, TNF‐α, Arg‐1, CD206, and IL‐10. “*,” *p* < 0.05; “**,” *p* < 0.01; “***,” *p* < 0.001; “****,” *p* < 0.0001.

The above results demonstrated that lactylation might be closely associated with neuroinflammation in SCI. Therefore, to verify the potential role of lactylation in modulating neuroinflammation, IFNγ and LPS were used to treat BV2 cells to simulate the secondary neuroinflammation after SCI in mice. Subsequently, lactate (Lac) was used to treat cells to promote lactylation. The experiments of lactylation showed that the lactylation was enhanced by IFNγ+LPS, which was further promoted by Lac or S‐Lac treatment as shown in the IFNγ+LPS + Lac group and the IFNγ+LPS + S‐Lac (sodium lactate) group (Figure 6A,B). In addition, compared to the control group, IFNγ+LPS treatment significantly elevated the lactate level, which was lower in the IFNγ+LPS + Lac group and the IFNγ+LPS + S‐Lac group (Figure 6C). To further explore the role of lactic acid in regulating neuroinflammation, the state of microglial polarization was assessed. The mRNA levels of M1 indicators (IL‐1β, iNOS, and TNF‐α) were elevated and the levels of M2 indicators (Arg‐1, CD206, and IL‐10) were reduced in the IFNγ+LPS group, indicating that inflammatory response was activated by IFNγ+LPS treatment (Figure 6D–I). Compared to the IFNγ+LPS group, the mRNA levels of IL‐1β, iNOS, and TNF‐α were significantly lower in the IFNγ+LPS + Lac group and the IFNγ+LPS + S‐Lac group, whereas the Arg‐1, CD206, and IL‐10 mRNA levels were remarkably higher in the IFNγ+LPS + Lac group and the IFNγ+LPS + S‐Lac group (Figure 6D–I), demonstrating that microglia pro‐inflammatory M1 polarization was suppressed and anti‐inflammatory M2 polarization was activated by LAC or S‐Lac treatment. The immunofluorescence results of iNOS showed that the elevated iNOS intensity by IFNγ+LPS treatment was lowered after the treatment of Lac or S‐Lac; however, the lowered CD206 intensity by IFNγ+LPS treatment was significantly raised by Lac or S‐Lac (Figure 6J–L). The above results showed that Lac or S‐Lac treatment could promote the M2 polarization of microglia and thus exerting anti‐inflammatory function. The protein levels of M1 indicators including IL‐1β, iNOS, and TNF‐α were elevated in the IFNγ+LPS group, which were significantly lowered by Lac or S‐Lac treatment (Figure 6M–P). The protein levels of Arg‐1, CD206, and IL‐10 in the IFNγ+LPS group were remarkably lower than those in the control group, which were significantly elevated by Lac or S‐Lac treatment (Figure 6M,Q–S).

### 3.7 Effects of exercise‐mediated production of lactate on the recovery motor function after SCI in mice

**FIGURE 7:**
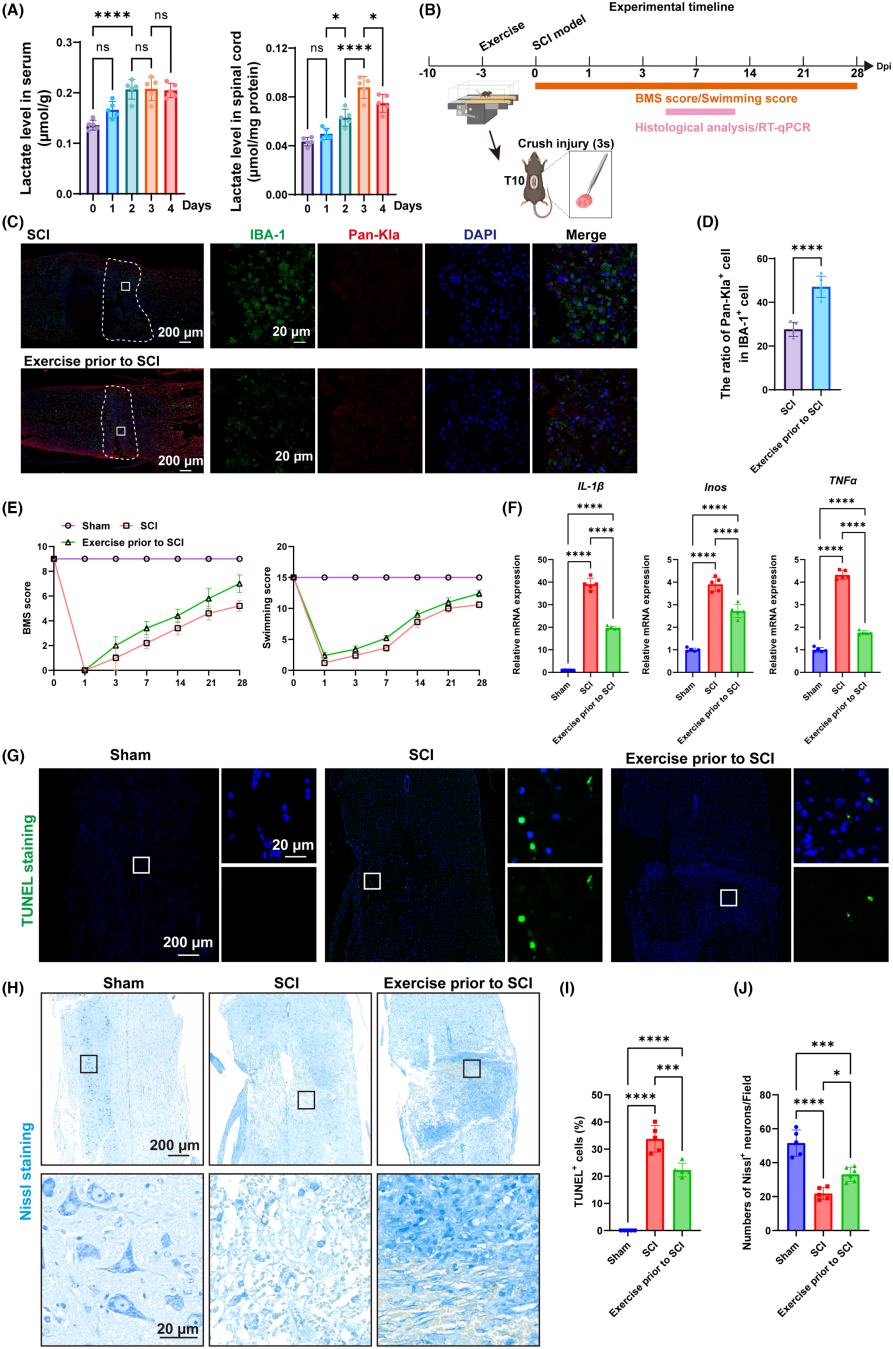
Effect of lactate on recovery of in mice. (A) Lactate level in serum and spinal cord of mice at different time points after running. (B) The study flow involving mice. (C) The immunofluorescence results of spinal cord. (D) Semiquantitative analysis of the immunofluorescence intensity of pan‐Kla. (E) BMS score of mice at different time points. (F) Swimming score of mice at different time points. (G,I) TUNEL staining and quantitative analysis of TUNEL^+^ cells. (H,J) Nissl staining and quantitative analysis of Nissl^+^ cells. “*,” *p* < 0.05; “**,” *p* < 0.01; “***,” *p* < 0.001; “****,” *p* < 0.0001.

According to previous studies, levels of lactate concentration rose with increased exercise intensity.^35^ The levels of lactate in serum and spinal cord of mice after running were detected. The results showed that the lactate levels in both serum and spinal cord tissue were significantly raised after daily‐running (Figure 7A). To investigate the role of lactate in regulating functional recovery and inflammatory microenvironment in spinal cord injury, mice were classified into the sham group, SCI group, and the SCI + exercise group. Mice in the SCI + exercise group underwent treadmill exercise for 3 days before SCI treatment. The study flow is shown in Figure 7B. The immunofluorescence results of spinal cord showed that the ratio of pan‐kla^+^ cells in IBA‐1^+^ cells in the SCI + exercise group was significantly raised when compared to the SCI group (Figure 7C,D). The motor function was evaluated using BMS score and swimming score. The results showed that both the BMS score and the swimming score were not significantly different at day one after SCI treatment (Figure 7E), indicating the consistency in the severity of SCI. The BMS score in the SCI + exercise group was significantly higher than that in the SCI group at days 14, 21, and 28(Figure 7E). In addition, compared to the SCI group, the SCI + exercise group showed higher swimming score at days 14, 21, and 28 (Figure 7E). At 3 days post‐injury, one centimeter of spinal cord tissue containing the central area of SCI was taken for mRNA extraction. The results showed that the SCI‐induced rase in mRNA levels of IL‐1β, iNOS, and TNF‐α were significantly lowered by exercise (Figure 7F). TUNEL staining was used to detect the apoptosis‐positive cells. Compared to the sham group, the SCI group showed significantly higher ratio of TUNEL‐positive cells, which was significantly restored in the SCI + exercise group (Figure 7G,I). Nissl staining was used to assess the number of neurons in the spinal cord and the results showed that SCI treatment reduced the number of neurons in spinal cord, which was significantly restored in the SCI + exercise group (Figure 7H,J).

## 4 DISCUSSION

The present study sought to elucidate the role of glycolysis‐derived lactate in modulating microglial function and attenuating spinal cord injury (SCI), with a specific focus on the molecular mechanisms underlying lactylation‐mediated neuroprotection. Our findings revealed that lactate, produced through increased glycolytic activity post‐SCI, serves not only as a critical energy source but also as a signaling molecule that influences the lactylation of microglia‐related proteins. This lactylation appears to play a pivotal role in dampening inflammatory responses and promoting tissue repair, highlighting its potential clinical significance for improving patient outcomes in SCI.

The relationship between SCI and metabolic reprogramming has gained considerable attention, as metabolic shifts are integral to the pathophysiological response following injury. Recent research has consistently shown that SCI induces a state of metabolic flux, particularly enhancing glycolytic pathways, which are crucial for meeting the augmented energy demands of damaged tissue and activated immune cells.^17^ Advanced studies have further elucidated the role of specific enzymes and metabolic intermediates that contribute to this glycolytic surge. For instance, the upregulation of glucose transporter proteins and key glycolytic enzymes such as hexokinase and lactate dehydrogenase has been documented, highlighting the cell's adaptive strategy to increase glucose uptake and fermentation, even under aerobic conditions, to sustain ATP production.^36^ These adaptive metabolic changes underscore the resilience of the spinal cord's response to injury and serve as potential therapeutic targets. By intervening in these pathways, it might be possible to ameliorate secondary injury processes and improve recovery. Moreover, recent interventions targeting metabolic modulators, such as 2‐deoxy‐D‐glucose, have shown promise in reducing glycolytic activity and improving neurological outcomes in preclinical models of SCI.^37^ These findings not only reinforce the potential of targeting metabolic pathways as a therapeutic strategy but also encourage the development of novel treatments that could inhibit pathological metabolism or enhance beneficial metabolic responses following SCI.

This present study contributes to the understanding of how glycolysis interacts with the inflammatory microenvironment in SCI. We found that increased glycolytic activity, particularly in microglial cells, correlates with elevated lactate production. Lactate, in turn, modifies the inflammatory response, suggesting a protective mechanism that counters the deleterious effects of SCI‐induced inflammation. These findings are consistent with an emerging literature that recognizes the role of metabolic pathways in regulating immune responses,^38^ thereby offering new insights into how manipulating these pathways could reduce inflammation and support recovery. Further research has demonstrated that lactate acts not merely as an energy source but also as a signaling molecule that can activate anti‐inflammatory pathways through its action on specific lactate receptors like GPR81, found on microglia and other immune cells.^39^ This receptor‐mediated signaling is crucial in shifting the immune response from a pro‐inflammatory to an anti‐inflammatory state,^40^ which is vital for recovery after SCI. Moreover, recent study has explored the role of pyruvate kinase M2, a key glycolytic enzyme, which apart from its metabolic functions, also participates in inflammatory signaling.^41^ This dual role underscores the complex interaction between metabolism and inflammation. Additionally, interventions that modulate glycolytic flux have been shown to affect the secretion of cytokines and chemokines,^42^ further substantiating the potential of targeting metabolic pathways to alter the immune landscape post‐SCI. This expanding body of knowledge deepens our comprehension of the metabolic‐immune interface in SCI and enhances the prospects for novel therapeutic strategies that address both metabolic dysregulation and excessive inflammation.

Furthermore, our results reinforce the emerging perspective of lactate as more than a metabolic byproduct in the context of SCI. The elevated levels of lactate following SCI not only reflect increased metabolic activity but also play crucial roles in modulating the inflammatory environment and facilitating repair processes. This is particularly significant in the context of SCI, where inflammation and tissue damage are predominant challenges. Recent research further substantiates this view,^43^ showing that lactate can directly influence the behavior of various immune cells, including macrophages and T cells, promoting a shift toward anti‐inflammatory and reparative phenotypes. Study indicated that lactate mediates these effects through specific mechanisms, such as the inhibition of pro‐inflammatory NF‐kB signaling pathways and the enhancement of anti‐inflammatory IL‐10 production.^44^ Additionally, lactate has been found to promote angiogenesis and collagen formation, processes that are vital for tissue repair and regeneration.^45^ By acting as a signaling molecule, lactate facilitates a microenvironment conducive to recovery, supporting cellular energetics and reducing oxidative stress. These multifaceted roles of lactate not only underscore its importance as a metabolic intermediary but also highlight its potential as a therapeutic agent that could be harnessed to improve outcomes after SCI by attenuating inflammation and enhancing tissue repair mechanisms.

Epigenetic modifications, including lactylation, play a crucial role in the pathophysiology of SCI. Changes in lactylation levels can affect chromatin structure and gene expression,^46^ thereby impacting the inflammatory response, glial scar formation, and neuroregeneration after SCI. The process of lactylation, influenced by the presence of lactate, is central to our findings. This post‐translational modification of proteins has been shown to have significant regulatory effects on cellular functions, particularly within immune cells.^47^ Our results suggest that enhancing lactylation could potentially be beneficial in treating SCI by modulating the inflammatory response and promoting repair mechanisms. Therefore, further research into the genes regulating lactylation could lead to novel gene therapy approaches that aim to enhance this modification, providing new avenues for SCI treatment. A previous study discovered histone lactylation and demonstrated its role in regulating gene expression during inflammation and repair.^24^ Recent advancements have identified specific enzymes responsible for the lactylation of histones, such as p300, which plays a crucial role in regulating gene expression through this modification.^48^ Study has demonstrated that lactylation of histones in immune cells leads to the transcriptional activation of anti‐inflammatory and reparative genes.^49^ In the present study, lactate administration was found to improve functional recovery, which might be mediated through lactylation of specific proteins involved in neuroprotection and repair. By identifying the key regulators of lactylation, researchers aim to manipulate this process to skew immune responses toward resolution of inflammation and promotion of healing in SCI. This line of inquiry not only broadens our understanding of lactate's role beyond its metabolic functions but also opens up promising therapeutic possibilities for managing the complex sequelae of SCI through epigenetic and metabolic interventions.

In the present study, several hub genes, including Fabp5, Lgals1, Vim, and Nefl, were confirmed to play significant roles in glycolysis and lactylation, which have important implications for SCI. Fabp5 primarily binds and transports fatty acids within cells, influencing glucose metabolism and glycolysis indirectly through its role in lipid metabolism.^50^ Although specific studies on Fabp5's role in lactylation are limited, its involvement in lipid metabolism suggests a potential link to metabolic reprogramming and cellular responses to lactate levels, which are critical in SCI pathology due to metabolic stress. Galectin‐1 (Lgals1), a β‐galactoside‐binding protein, influences glycolytic enzymes and pathways, potentially modulating glycolysis through its regulatory roles.^51^ Lgals1 may also be involved in lactylation by regulating cellular responses to metabolic changes and stress, which are common after SCI, thereby modulating protein–protein interactions and signaling pathways. Vimentin (Vim), an intermediate filament protein, interacts with glycolytic enzymes, suggesting a role in organizing and regulating glycolysis within the cytoskeleton. Vim may also be subject to lactylation, influencing its function and stability, impacting cell migration, proliferation, and stress responses—processes that are crucial in the repair and regeneration of spinal cord tissues.^52^ Neurofilament light chain (Nefl), a component of the neurofilament protein complex in neurons, influences cellular energy metabolism and homeostasis, potentially affecting glycolytic activity indirectly.^53^ Nefl could also be involved in lactylation, impacting its structural and functional roles in neurons, with implications for neurodegenerative diseases and neuronal metabolism. Understanding the involvement of these hub genes in glycolysis and lactylation provides insights into the molecular mechanisms underlying SCI and may reveal potential therapeutic targets for improving recovery and regeneration after SCI.

Despite these promising findings, our study has limitations. The complexity of the metabolic pathways involved and the multifactorial nature of SCI mean that the direct application of these findings to clinical settings requires cautious interpretation and further validation. The precise mechanisms through which lactylation affects cellular behavior in the SCI context remain to be fully elucidated. Furthermore, the variability in injury severity and locations, commonly seen in clinical populations, was not fully addressed in our homogeneous animal samples. This could affect the generalizability of our findings to diverse patient groups. Another limitation is the potential off‐target effects of enhancing lactylation, which could impact other metabolic or cellular pathways, leading to unforeseen complications.

## 5 CONCLUSIONS

In conclusion, utilizing single‐cell RNA sequencing, we mapped the cellular and molecular changes post‐injury, highlighting an increase in glycolytic enzymes and lactate within microglial cells. These changes correlated with alterations in gene expression that enhance protein lactylation. Our in vivo and in vitro experiments showed that increasing lactate production can reduce inflammation and aid recovery in SCI models, illustrating how lactate‐modulated lactylation influences microglial phenotypes and inflammatory mediator production. This research advanced our understanding of SCI metabolic responses and suggested that targeting metabolic pathways and lactylation could improve SCI treatment outcomes. Future research and clinical trials will be crucial to develop these findings into effective therapies, aiming to enhance the quality of life for SCI patients.

## AUTHOR CONTRIBUTIONS

XFS, KQS, and QJK designed this study. BZ, FDL, YYS, and CLJ conducted the experiments. Data analysis was carried out by KQS, FDL, and CLJ. The manuscript was drafted by FDL and KQS. All authors read and approved the final version of the manuscript.

## FUNDING INFORMATION

This study was supported by the National Natural Science Foundation of China (Grant No. 82172381, 82302760), the Shanghai Shenkang Project (Grant No. SHDC2020CR1024B), and the Peizhi Project of Changzheng Hospital (2020YCGPZ‐106).

## CONFLICT OF INTEREST STATEMENT

We declare that we have no conflict of interest.

## Supporting information


FigureS1‐S2

## Data Availability

The data that support the findings of this study are available from the corresponding author upon reasonable request.
