# Supplementary material for: Single‐cell RNA sequencing integrated with bulk RNA sequencing analysis reveals the protective effects of lactate‐mediated lactylation of microglia‐related proteins on spinal cord injury
Source: CNS Neurosci Ther. 2024 Sep 1;30(9):e70028. doi: 10.1111/cns.70028 (PMC11366449; doi:10.1111/cns.70028)
Supplement: Supplementary file 1 — FigureS1‐S2 [file CNS-30-e70028-s001.docx]

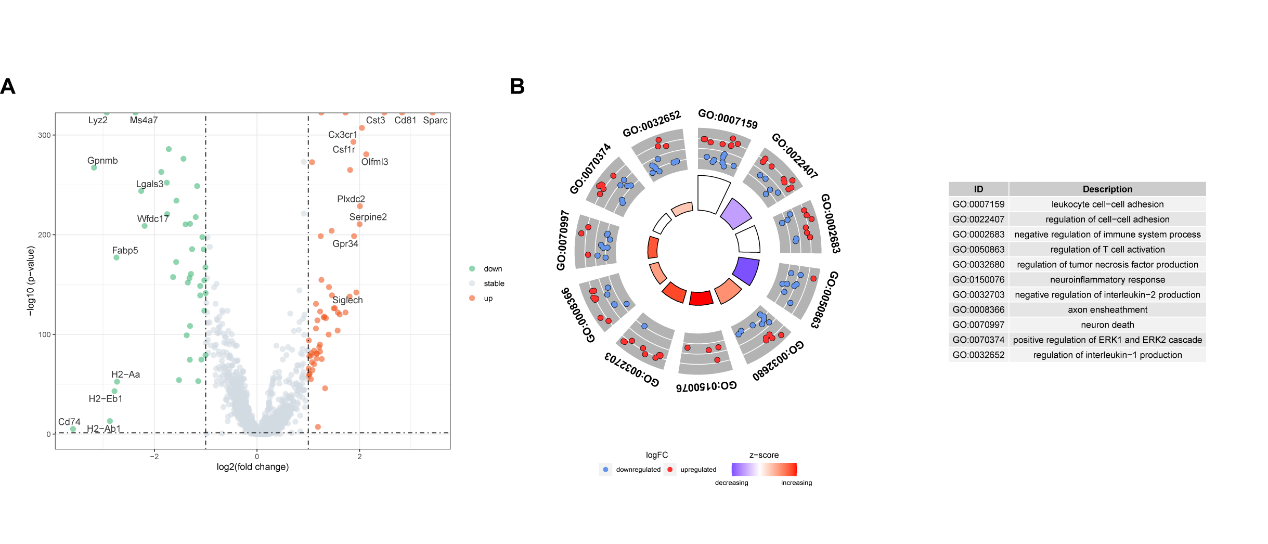


**Figure S1. The differentially expressed genes and GO enrichment.** (A) Volcano plot of differentially expressed genes in various clusters. (B) The GO enrichment results.


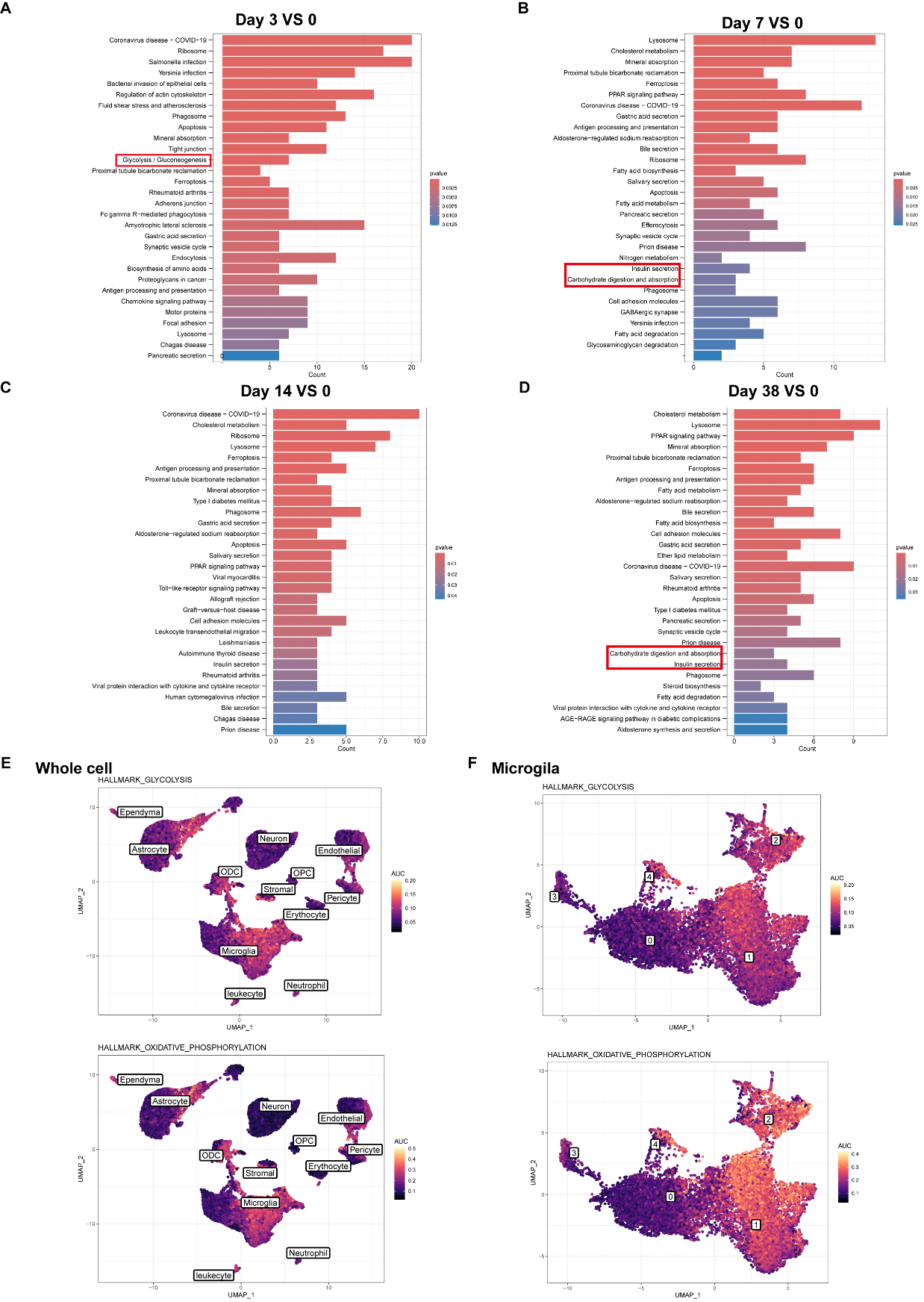


Figure S2. After spinal cord injury, microglia showed an imbalance of global glycolytic homeostasis. (A) The KEGG enrichment results between samples at day 3 and day 0. (B) The KEGG enrichment results between samples at day 7 and day 0. (C) The KEGG enrichment results between samples at day 14 and day 0. (D) The KEGG enrichment results between samples at day 38 and day 0. (E) The UMAP analysis results of glycolysis and oxidative phosphorylation score for whole cells. (F) The UMAP analysis results of glycolysis and oxidative phosphorylation score for microglia.
